# Characterizing a region on BTA11 affecting β-lactoglobulin content of milk using high-density genotyping and haplotype grouping

**DOI:** 10.1186/s12863-017-0483-9

**Published:** 2017-02-22

**Authors:** Nicolas Bedere, Henk Bovenhuis

**Affiliations:** 1 Animal Breeding and Genomics Centre, Wageningen University, P.O. Box 338, 6700 AH Wageningen, The Netherlands; 2 Present address: PEGASE, Agrocampus Ouest, INRA, 35590 Saint-Gilles, France

**Keywords:** Dairy cow, Bovine, β-lactoglobulin, Haplotype, Association study

## Abstract

**Background:**

Milk β-lactoglobulin (β-LG) content is of interest as it is associated with nutritional and manufacturing properties. It is known that milk β-LG content is strongly affected by genetic factors. In cattle, most of the genetic differences are associated with a chromosomal region on BTA11, which contains the β-LG gene. The aim of this study was to characterize this region using 777 k SNP data (BovineHDbeadChip) and perform a haplotype-based association study. A statistical approach was developed to build haplotypes that capture the genetic variation associated with this genomic region.

**Results:**

The SNP with the most significant effect on β-lactoglobulin content was one of the 2 causal mutations responsible for the β-lactoglobulin protein variants A/B. Haplotypes based on 2 to 5 selected lead SNP were clustered in groups with different effects on β-lactoglobulin content. Four different groups were identified suggesting that β-lactoglobulin variant A and B can be further refined in A_1_, A_2_, B_1_ and B_2_.

**Conclusions:**

This study showed that β-lactoglobulin protein variants A/B do not explain all genetic variation associated with the tail part of BTA11 but this region contains more than one mutation with an effect on β-lactoglobulin content. These findings can be used for selection of cows with higher cheese yield, which is desirable for the dairy industry.

## Background

Bovine milk contains around 3–4% protein, which consists of caseins and whey proteins. Around 80% of the milk proteins are caseins and the remaining fraction is made up of soluble proteins of which β-lactoglobulin (β-LG) is the most important [1, 2]. β-LG is of interest as it is associated with nutritional and manufacturing properties of milk. Interestingly, human milk does not contain β-LG and, therefore, β-LG may be less important for human infants. Some people are oversensitive to milk protein (cow’s milk allergy) and β-LG has been considered as a major milk allergen [3]. This was one of the reasons for selecting a cow with milk lacking β-LG [4]. On the other hand, β-LG is a rich source of essential amino acids and has therefore a high nutritional value [5].

Two distinct forms of the β-LG protein (A and B) were described in 1955 [6] and several studies have shown relations between protein variants A and B of β-LG, cheese yield and heat stability of milk [7, 8]. Milk from cows homozygous for β-LG protein variant B results in approximately 3% more cheese as compared to milk from cows homozygous for β-LG protein variant A [7]. Further, milk with β-LG protein variant B results in a lower fouling rate of heating equipment [9] and therefore in lower costs of cleaning heating equipment.

Milk β-LG content is strongly affected by genetic factors: 80% of the differences are due to genetics [10]. A genome wide association study identified a chromosomal region on BTA11 with a major effect on β-LG content [11]. This region contains the β-LG gene which codes for the β-LG protein. Several studies showed that β-LG protein variants A and B are associated with β-LG content in the milk: the β-LG B variant is associated with a lower β-LG content [12–14]. Schopen et al. [11] found that after adjusting for the effects of β-LG protein variants a significant proportion of the genetic variance remains associated with the chromosomal region on BTA11. This suggests that the mutations responsible for the differences between β-LG A/B protein variants are not the causal mutations or that this region contains multiple mutations with an effect on β-LG content. The recent availability of high density (777 k) SNP array enables to fine map the targeted region on BTA11 and investigate if one or multiple mutations are responsible for the observed effects. In addition, defining haplotypes that capture all genetic variation in β-LG content associated with this region will allow more efficient selection for β-LG content than would be possible based on β-LG protein variants.

This study aims to fine map the chromosomal region on BTA11 associated with β-LG content using 777 k SNP data and to investigate if one or multiple mutations are responsible for the observed effects.

## Results

The average protein content of the milk samples was 3.50% (w/w%) and 8.34% of the protein consists of β-LG (data not shown). Table 1 shows the estimated variance components and ratios for β-LG content (unadjusted for SNP effects). The estimated heritability for β-LG content is 0.78 and the proportion of the variation explained by differences between herds is 0.05.

**Table 1:** Variance components (herd variation, polygenic additive genetic variation and residual variation), intra-herd heritability and proportion of variance due to herd for the un-adjusted β-LG content (wt/wt%) and the adjusted β-LG contents

	Un-adjusted β-LG content	Adjusted β-LG content				
		β-LG^1^	β-LG^2^	β-LG^3^	β-LG^4^	β-LG^5^
σ^2^_herd_	0.08	0.10	0.11	0.10	0.10	0.10
σ^2^_a_	1.121	0.111	0.090	0.090	0.086	0.079
σ^2^_e_	0.31	0.23	0.23	0.23	0.23	0.24
h^2^	0.78	0.33	0.28	0.28	0.27	0.25
h_herd_	0.05	0.23	0.25	0.24	0.24	0.24

Un-adjusted β-LG content is the β-lactoglobulin content as fraction of the total protein fraction

β-LG^1^ is β-LG adjusted for the genotype of the cows for Q-Tag SNP_1_

β-LG^2^ is β-LG^1^ adjusted for the genotype of the cows for Q-Tag SNP_2_

β-LG^3^ is β-LG^2^ adjusted for the genotype of the cows for Q-Tag SNP_3_

β-LG^4^ is β-LG^3^ adjusted for the genotype of the cows for Q-Tag SNP_4_

β-LG^5^ is β-LG^4^ adjusted for the genotype of the cows for Q-Tag SNP_5_

### Single SNP association

**Fig. 1:**
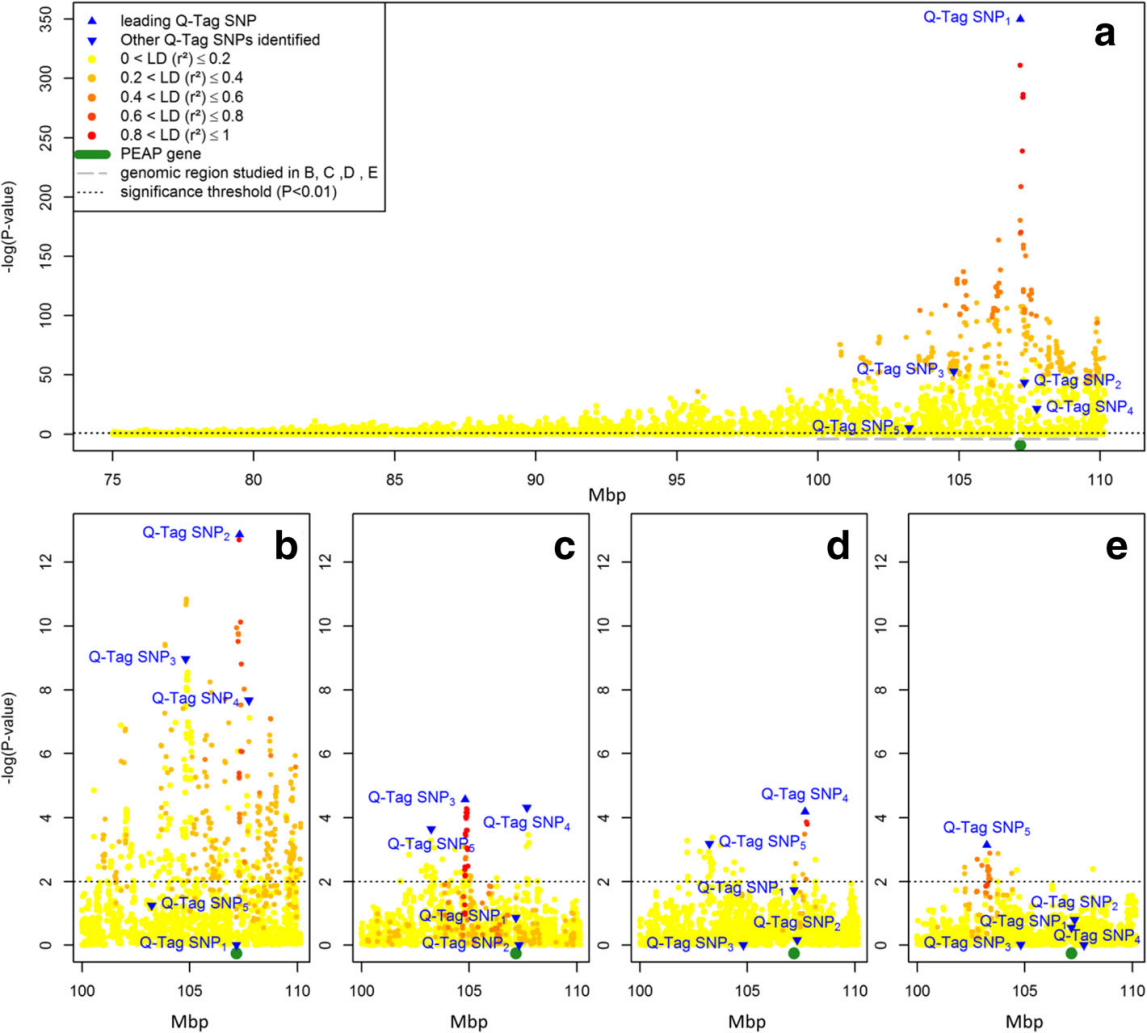
Association study plots for milk β-LG content. Plot of -log_10_(*P*-values) on the y-axis for the association of 9,053 SNPs located on the tail part of the *Bos taurus* autosome 11, positions based on Btau 4.2 (x-axis), with un-adjusted milk β-LG content (**a**). Three -log_10_(*P*-values) estimated as infinite were set equal to a value of 350. The colour gradient represents the LD (r^2^) between the Q-Tag SNP and the other SNPs. Plots of -log_10_(*P*-values) for the association of 2,584 SNPs located in the region of interest (underlined in *grey* **a**) with milk β-LG content adjusted for the genotype of Q-Tag SNP_1_ (**b**), and Q-Tag SNP_2_ (**c**), and Q-Tag SNP_3_ (**d**), and Q-Tag SNP_4_ (**e**)

Figure 1 shows the –log_10_(*P*-values) of the association between SNP located on the tail part of BTA11 and the unadjusted β-LG (Fig. 1.a) content and β-LG content adjusted for the effects of one or multiple Q-Tag SNP (Fig. 1.b-e). Figure 1.a shows the results for 9,053 SNP located between 75 Mb and 110 Mb on BTA11. The lead SNP had a highly significant effect and a –log_10_(*P*-value) >350. The lead SNP, i.e. Q-Tag SNP_1_ (*rs110066229*) is located in the third exon of β-LG gene (*PAEP*) and is one of the 2 mutations responsible for the difference between β-LG protein variants A and B [15]. The colour gradient in Fig. 1.a shows the Linkage Disequilibrium (LD as quantified by the r^2^) between Q-Tag SNP_1_ and the other SNP. Table 1 shows that after adjusting β-LG content for the effect of Q-Tag SNP_1_, the additive genetic variance drops from 1.121 to 0.111 (i.e. when analysing the trait β-LG^1^). In other words, Q-Tag SNP_1_ explained 91% of the additive genetic variation of the unadjusted β-LG content. Herd variation slightly increased after adjusting for the effect of Q-Tag SNP1 and as a consequence of the decrease in additive genetic variation, the part of phenotypic variation explained by the herd variation increased. Figure 1.b shows the significance for 2,584 SNP located between 100 Mb and 110 Mb on BTA11 for β-LG^1^. Q-Tag SNP_2_ (*rs110144148*) had a highly significant effect on β-LG^1^ with a –log_10_(*P*-value) of 12.9. This shows that not all variation associated with this chromosomal region is captured by the difference between the β-LG protein variants A and B. Q-Tag SNP_2_ is located at 107.3 Mb, i.e. distal from the *PAEP* gene. The additive genetic variation is further reduced after adjusting for Q-Tag SNP_2_ to 0.090, i.e. 8% of additive genetic variance of the unadjusted β-LG content (Table 1). Figure 1.c shows the results of the association study for β-LG^2^. Q-Tag SNP_3_ (*rs136463816*) is located at 104.8 Mb and has a –log_10_(*P*-value) of 4.6. The additive genetic variation for β-LG^3^ is 0.090. Figure 1.d shows the significance of the SNP for β-LG^3^. Q-Tag SNP_4_ (*rs136800235*) is located at 107.7 Mb and has a –log_10_(*P*-value) of 3.86. Figure 1.e shows the results of the association studies with β-LG^4^. Q-Tag SNP_5_ (*rs17871095*) is located at 103.2 Mb and has a –log_10_(*P*-value) of 3.14. More details about the Q-Tag SNP can be found in Table 2.

**Table 2:** Information on the Q-Tag SNP identified in this study

Name given^a^	dbSNP ID^b^	MAF^c^	Position (based on Btau 4.2)
Q-Tag SNP_5_	rs17871095	0.15	103226704
Q-Tag SNP_3_	rs136463816	0.44	104803861
Q-Tag SNP_1_	rs110066229	0.38	107168524
Q-Tag SNP_2_	rs110144148	0.27	107312422
Q-Tag SNP_4_	rs136800235	0.18	107749128

^a^subscript indicates the order of the Q-Tag SNP in the stepwise procedure

^b^ID given on the dbSNP of NCBI (http://www.ncbi.nlm.nih.gov/)

^c^Minor Allele Frequency

### Haplotype effects

**Fig. 2:**
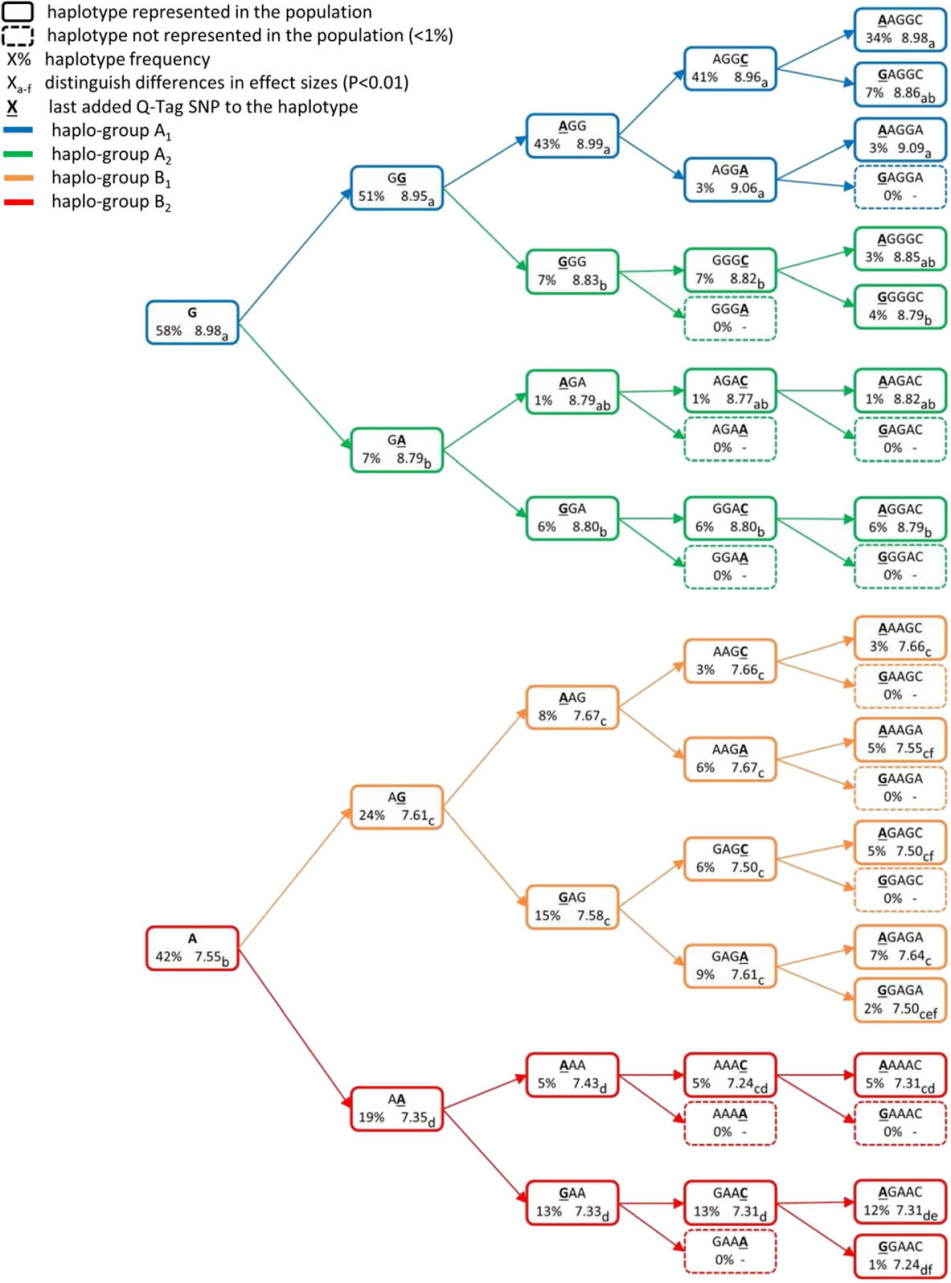
Tree of haplotypes and haplo-groups. Haplotypes are constructed based on different numbers of SNPs. SNPs that make up the haplotype are ordered according their map position (Btau 4.2). Last added SNP is underlined. Percentages are haplotypes frequencies in the population and numbers are predicted effects of carrying one haplotype. Differences between predicted haplotype effects (constructed based on the same number of SNP) are tested and effect sizes with different letters differ significantly (*p* < 0.01). Dashed line represents haplotypes which are at a frequency lower than 1%

Figure 2 shows a tree of the haplotypes that were constructed based on Q-Tag SNP, the haplotype frequencies, and the predicted haplotype effects on β-LG content. For comparison; the predicted allelic effects of Q-Tag SNP_1_, i.e. the β-LG protein variants (β-LG A corresponds to the G allele of the SNP and β-LG variant B corresponds to the A allele of the SNP) are also shown in Fig. 2. The predicted β-LG content of cows carrying one copy of the G allele (β-LG A) is 8.98 (w/w%) and for cows carrying one copy of the A allele (β-LG B) this is 7.55 (w/w%). Having one copy of the β-LG protein variant A thus results in a 1.43% higher β-LG content as compared to having β-LG protein variant B. This corresponds to a difference between AA and BB (i.e. having two copies) of 2.86% (Table 3).

**Table 3:** Variance components (additive genetic variation, haplo-group variation and residual variation), proportion of variance due to haplo-groups^a^ and estimated effects of haplo-groups on β-LG content and their distribution in the population

	Haplotype based on				
	Q-Tag SNP_1_	2 Q-Tag SNP	3 Q-Tag SNP	4 Q-Tag SNP	5 Q-Tag SNP
σ^2^_a_	0.113	0.082	0.084	0.084	0.084
σ^2^_haplo-group_	1.022	0.664	0.685	0.685	0.685
σ^2^_e_	0.288	0.297	0.293	0.293	0.293
h^2^_haplo-group_	0.718	0.637	0.645	0.645	0.645
	Estimated effect^b^				
haplo-group A_1_^a^	0.71 (58%)	0.78 (51%)	0.79 (44%)	0.79 (44%)	0.79 (44%)
haplo-group A_2_		0.62 (7%)	0.62 (14%)	0.62 (14%)	0.62 (14%)
haplo-group B_2_		−0.57 (24%)	−0.58 (24%)	−0.58 (24%)	−0.58 (24%)
haplo-group B_1_	−0.71 (42%)	−0.83 (18%)	−0.84 (18%)	−0.84 (18%)	−0.84 (18%)
Difference A_1_A_1_-B_1_B_1_	2.86	3.22	3.26	3.26	3.26

^a^Haplotypes are assigned to one of the 4 haplo-groups as described in Fig. 2

^b^Effect of one copy of the haplotype with frequency in the population in parentheses

When considering Q-Tag SNP_1_ and Q-Tag SNP_2_, haplotypes GG, GA, AG and AA can be distinguished. Pairwise comparisons of the predicted haplotype effects show that all four haplotypes have significantly different effects on β-LG content. Haplotype GG has the strongest positive effect on β-LG content and its effect is significantly different from haplotype GA, which also is associated with an increase of β-LG content. The GG and the GA haplotypes differentiate among β-LG protein variant A and will therefore be referred to as A_1_ (GG) and A_2_ (GA). The two other haplotypes AG and AA differentiate β-LG protein variant B and will be referred to as B_2_ (AG) and B_1_ (AA). These two haplotypes are associated with lower β-LG content. The estimated difference between haplotype GG and AA is 1.60% and therefore the expected difference between cows carrying two copies of haplotype GG versus those who have two copies of haplotypes AA is 3.20%.

Further refining the haplotypes by considering three Q-Tag SNPs results in one haplotype (AGA) which occurs at a very low frequency in the population (1%). Adding the third Q-Tag SNP does result in a further refinement: the GG haplotype, which was assigned to haplo-group A_1_, is differentiated in AGG and GGG haplotypes which have significantly different effects. Whereas the AGG haplotype is assigned to haplo-group A_1_, the GGG haplotype is assigned to haplo-group A_2_.

When considering 4 or 5 Q-Tag SNP to build haplotypes, there is an increasing number of haplotypes that occur at low frequencies in the population. The predicted effects of haplotypes with frequencies smaller than 1% were not included in Fig. 2. Haplotypes consisting of 4 or more Q-Tag SNP could not always be unequivocally assigned to one of the four haplo-groups, e.g. the predicted effect of haplotype AGAC (haplo-group A_2_) is not significantly different from effects of haplotypes in haplo-group A_1_ and the predicted effect of haplotype AAAC (haplo-group B_1_) is not significantly different from effects of haplotypes in haplo-group B_2_.

Table 3 shows the estimated variance components, genetic parameters and estimated effects for the haplo-groups. Haplotypes were assigned to one of the four haplo-groups as is shown in Fig. 2. For comparison, results are also shown for a situation when considering only one Q-Tag SNP, i.e. modelling the allelic effects of the β-LG protein variants as a random effect. Results show that haplotype variance increases from 0.664 to 0.685 when moving from 2 to 3 Q-Tag SNP whereas the residual polygenic additive genetic variation tends to decrease (0.297–0.293). Adding more than 3 Q-Tag SNP did not further increase the variance explained by the haplo-groups. The proportion of the variance explained by differences among haplo-groups was 63.7% when considering 2 Q-Tag SNP and increased to 64.5% when haplotypes were based on 3 or more Q-Tag SNP. In addition, the difference of estimated effect size on β-LG content between individuals homozygous for haplo-group A_1_ (A_1_A_1_) and individuals homozygous for haplo-group B_1_ (B_1_B_1_) increased from 2.86 for considering only one Q-Tag SNP to 3.26 when considering 3 or more Q-Tag SNP. The analyses indicate that 89% of the additive genetic variation in β-LG content can be explained by the genomic region between 100 Mb and 110 Mb of BTA11.

## Discussion

β-LG is a milk protein which is the product of the *PAEP* gene. Therefore it is expected that the phenotype-genotype relationship of milk β-LG content is relatively simple. The heritability of milk β-LG content was estimated to be 0.80 indicating that differences in β-LG content are strongly determined by genetic factors [10]. A genome wide association study indicated that a chromosomal region on BTA11 containing *PAEP* explains most of the genetic variation in β-LG content [11]. However, after adjusting for β-LG protein variants, a significant proportion of the genetic variance remains associated with this genomic region. The authors found another SNP that significantly explained 1.5% of the genetic variance in the region after adjusting for the effect for protein variants. This suggests that mutations responsible for the differences between β-LG A/B protein variants are either not causal or that there are multiple mutations in this chromosomal region with an effect on β-LG content. In the current study we defined haplotypes based on Q-Tag SNP and using this approach the genetic variation associated with a chromosomal region can be captured based on a relatively small number of SNP. The haplotypes were clustered in 4 groups, A_1_, A_2_, B_1_ and B_2_, with distinct effects on β-LG content suggesting that this chromosomal region contains more than one mutation with an effect on β-LG content.

### Fine mapping using 777 k SNP array

Fine mapping the genomic region between 75 and 110 Mb on BTA11 using the high density SNP array (777 k) resulted in a substantial increase in SNP density as compared to the 50 k array SNP panel. Therefore, the high density SNP array is expected to increase the probability of finding SNP in strong Linkage Disequilibrium (LD) with the causal mutation(s). However, the lead SNP based on the 777 k array (Q-Tag SNP_1_) is identical to the lead SNP based on the 50 k array [11]. Q-Tag SNP_1_ is one of the 2 causal mutations for β-LG protein variants A/B [15]. Several studies, in different breeds and populations, have consistently shown associations between β-LG protein variant A and increased β-LG content [12, 14, 16]. This suggests that Q-Tag SNP_1_ actually may be one of the causal mutations or at least located close to the causal mutation. Q-Tag SNP_1_ explains most but not all of the additive genetic variation associated with this genomic region. This suggests that either the causal mutation has not been identified or that this region contains multiple mutations with an effect on β-LG content.

### Haplotype construction and associations

The use of haplotypes in genome-wide association studies has been suggested because they may be in stronger LD with the Quantitative Trait Loci (QTL) than single SNP and therefore may have increased power to detect QTL [17, 18]. The advantage of haplotype over single SNP association study is expected to be smaller for high density as compared to low density SNP arrays. However, QTL with low Minor Allele Frequencies (MAFs) may be in low LD with SNP present on the SNP array due to ascertainment bias. In addition, single SNP may not be able to capture all genetic variation associated with a genomic region, e.g. because a region contains multiple causative mutations. Haplotypes provide a more detailed characterization of a region and can be used for dissecting effects associated with a genomic region.

An important difficulty with a haplotype-based approach is that the number of haplotypes becomes very large when haplotypes are based on an increasing number of SNP. E.g. when haplotypes are constructed based on the lead SNP and 10 adjacent SNP (5 on each side) 13 haplotypes are segregating in the current data and when 20 adjacent SNP are used (10 on each side of the lead SNP) the number of haplotypes is 53. Having a large number of haplotypes reduces the number of observations per haplotype: several haplotypes have frequencies smaller than 0.1%. The small number of observations per haplotype will likely dilute association signals. Construction haplotypes based on Q-Tag SNP strongly limits the number of possible haplotypes while still capturing the variation associated with a region. However, even when building haplotypes on Q-Tag SNP, the number of haplotypes is 2^n^ where n is the number of Q-Tag SNP.

Haplotypes can be considered as alleles of a single multi-allelic marker and as such can be used in an association. The maximum number of genotype effects of this “super” marker is ½ m(m + 1) where m is the number of haplotypes (or alleles of the “super” marker). For example, for 8 haplotypes there are at maximum 36 effects to be estimated which is a further risk of diluting association signals. Therefore, we restricted the number of effects to be estimated by assuming additivity of the haplotype effects. The design matrices of both haplotypes were combined and the statistical analysis results in one estimated haplotype effect.

Even when using the described approach, inevitably a few common and several rare haplotypes will appear when the number of Q-Tag SNP increases (Fig. 2). These low frequency haplotypes may have a unique effect but it is not possible to significantly distinguish their effects from the effect other haplotypes. The current study shows that based on 3 Q-Tag SNP most of the additive genetic variation associated with this genomic region can be captured. Indeed, the additive genetic variation is about 1.121 for unadjusted β-LG content and 0.084 for haplotypes based on 3 Q-Tag SNP (i.e. a reduction of 93%). Any additional refining of haplotypes did not increase the genetic variation explained by the haplotypes. Adding Q-Tag SNP increases the number of haplotypes but in general decreases the number of cows carrying copies of a specific haplotype. This decreases the power of unequivocally assigning haplotypes to haplotype groups or to identify new haplotype groups with distinct effects.

### Effects of haplotypes

In the current study we were able to identify 4 groups of haplotypes with distinct effects on β-LG content: A_1_, A_2_, B_1_ and B_2_. This is consistent with other study suggesting that the genetic variant A and B of *PAEP* can be further refined into 4 genetic variants in total through splitting both the A and B variants into 2 sub-variants [19]. Both the SNPs identified in this study and the haplotypes constructed are different from the one of the present study although closely located and possibly linked with the same causal mutations. Effects of haplotypes at low frequency cannot be predicted very accurately and therefore complicates assigning them unequivocally to one of the existing haplo-groups. The results suggest that the number of haplotype groups with distinct effects does not increase beyond the already existing four when haplotypes are based on three Q-Tag SNPs. However, further refinement of the haplo-groups did take place: haplotype GG, which was assigned to haplo-group A_1_, was split in haplotype AGG which was assigned to haplo-group A_1_ and haplotype GGG which was assigned to haplo-group A_2_.

Having more than two groups of haplotypes suggests that this chromosomal region contains more than one mutation with an effect on β-LG content. Assuming that there are two mutations underlying the observed haplotype effects, i.e. locus 1 and locus 2, than haplo-group A_1_ carries a “+” allele at locus 1 and a “+” allele at locus 2, haplo-group A_2_ carries a “+”allele at locus 1 and a “-” allele at locus 2, haplo-group B_2_ carries a “-” allele at locus 1 and a “+” allele at locus 2 and haplo-group B_1_ carries a “-”allele at locus 1 and a “-” allele at locus 2. Using the results from Table 3 (based on 3 Q-Tag SNP), the estimated additive effect (i.e. “a” in Falconer notation) at locus 1 is 1.42 and 0.22 at locus 2. The estimated frequencies of the alleles which increase β-LG content are 0.58 at locus 1 and 0.66 at locus 2.

β-LG protein variants are not associated with protein content of milk but are strongly associated with the casein index [14]. When analysing the haplo-groups, we also do not find an effect on milk protein content (h^2^_haplo-group_ = 0.00) but there is a large effect on the casein index (h^2^_haplo-group_ = 0.57). The estimated effect on the casein index is −0.87 for haplo-group A_1_, −0.69 for haplo-group A_2_, 0.62 for haplo-group B_2_ and 0.94 for haplo-group B_1_ (haplo-groups based on 3 Q-Tag SNP). The difference in casein index between the β-LG protein variants BB and AA is 3.15% whereas the difference between extreme haplotype groups (B_1_B_1_ vs. A_1_A_1_) is 3.63%. The casein index is directly related to the efficiency of cheese production and therefore selecting for B_1_B_1_ is beneficial to the dairy industry.

In order to find the causal mutations a possible next step is to sequence animals. The haplotypes can be used to design sequencing studies and individuals from different haplo-groups can be identified for sequencing (e.g. A_1_A_1_ versus B_1_B_1_). Although knowledge on the causal mutations is currently lacking, the identified haplotypes can be used in selection.

## Conclusions

The lead SNP from the single SNP association using the high density SNP array is one of the 2 mutations responsible for the difference between β-LG protein variants A and B. The statistical approach developed can be used in fine mapping, haplotypes reconstruction and association studies with quantitative traits. A tool enabling to decide at which step to stop the stepwise association study has to be found. We constructed haplotypes based on 2 to 5 Q-Tag SNP and clustered in groups with significantly different effects on β-LG content. This study showed there are 4 different haplo-groups: A_1_, A_2_, B_1_ and B_2_ (named by analogy to protein variants A and B). The existence of more than two groups of haplotypes suggests that this chromosomal region contains more than one mutation with an effect on β-LG content. These findings can be used for selection of cows with higher cheese yield.

## Methods

### Population

The present study was part of the Dutch Milk Genomics Initiative. In this project Milk samples were collected from 1,713 primiparous cows on 383 commercial herds. These cows descended from one of five proven bulls representing five large half-sib families (782 cows), one of 50 test bulls representing 50 small half-sib families (760 cows), or from 15 other proven bulls (171 cows). In the last group of 171 cows, at least 3 cows per herd were sampled. The pedigrees of the cows were supplied by the CRV (Arnhem, The Netherlands). Each cow was at least 87.5% Holstein-Friesian. The average age of the cows at first calving was 2.1 years and the cows calved between June 2004 and February 2005. Almost all the same animals were used in previous studies for the genetic analysis of milk protein [10, 11].

### Phenotypes

Morning milk samples, collected between February and March 2005 on 1,713 Dutch Holstein-Friesian cows, were analysed for detailed milk protein composition. The β-LG content was determined by Capillary Zone Electrophoresis (CZE) as described by Heck et al. (2008). Protein content (wt/wt%) was predicted based on infrared spectroscopy by routine milk recording (for more details see [20]).

### Genotypes

DNA for genotyping was isolated from blood samples of 1,736 cows. A 50 k SNP chip developed by CRV (cooperative cattle improvement organization, Arnhem, the Netherlands) was used to genotype cows as well as the sires of the cows using the Infinium assay (Illumina, USA) [11]. In addition, 55 of the sires of these cows were genotyped with the BovineHDbeadChip (about 777 k, Illumina, USA). For imputing the 1,736 cows from 50 to 777 k a reference population of 1,333 Dutch Holstein-Friesian cows was available. The reference population included the 55 sires. Other animals in the reference population were provided by CRV. For imputation and phasing BEAGLE 3.3 was used [21]. In a first step, the consistency of genotypes between parents and offspring was assessed. The pedigree was assumed to be correct if less than 0.5% of the homozygous markers in the offspring were not in agreement with the parental genotype. In a second step, 777 k SNP genotypes were imputed and phased for all 1,736 cows using information of the 50 k SNP genotypes of all animals and the 777 k SNP genotypes of animals in the reference population [22].

The genotypes of the 2 SNP responsible for the amino acid changes in the β-LG variants A and B and 8 other SNP associated with β-LG content [15] were available for 1,611 cows. For 125 cows these SNP genotypes were missing and imputed and phased using BEAGLE 3.3 [22]. The positions of the SNP were based on the Btau 4.2 assembly.

In total, 1,647 cows had both phenotypic and genotypic information and were used for the association study. Based on previous results [11], we focused on the region from 75 Mb to 110 Mb on BTA11 in the current study. In that region, 9,925 SNP genotypes were available of which 872 SNP were homozygous in our population and therefore not included in the association study.

### Statistical analyses

The single SNP association study was performed using the following model:

1 $$ \begin{array}{c}\hfill {\mathrm{y}}_{\mathrm{k}\mathrm{lmno}}=\upmu +{\upbeta}_1\mathrm{di}{\mathrm{m}}_{\mathrm{k}\mathrm{lmno}}+{\upbeta}_2{\mathrm{e}}^{-0.05\mathrm{di}{\mathrm{m}}_{\mathrm{k}\mathrm{lmno}}}+{\upbeta}_3\mathrm{c}{\mathrm{a}}_{\mathrm{k}\mathrm{lmno}}+{\upbeta}_4\mathrm{ca}{2}_{\mathrm{k}\mathrm{lmno}}+\mathrm{seaso}{\mathrm{n}}_{\mathrm{k}}\hfill \\ {}\hfill +\mathrm{scod}{\mathrm{e}}_{\mathrm{l}}+{\mathrm{SNP}}_{\mathrm{m}}+\mathrm{anima}{\mathrm{l}}_{\mathrm{n}}+\mathrm{her}{\mathrm{d}}_{\mathrm{o}}+{\mathrm{e}}_{\mathrm{k}\mathrm{lmno}}\hfill \end{array} $$

where y_klmno_was the β-LG content, μ is the mean for β-LG content, dim_klmno_ is the covariate describing the effect of the numbers of days in milk, modelled with Wilmink curve [23] as explained in Heck et al. (2008) [14], ca_klmno_ is the covariate describing the effect of the age at first calving as linear and quadratic, season_k_ is the fixed effect calving season (k = 1, 2 or 3), scode_l_ is the fixed effect of sire group (l = 1, 2 or 3), SNP_m_ is the fixed effect of the SNP, animal_n_ is the random additive genetic effect of the animal n, herd_o_ is the random herd effect and e_klmno_ is the random residual effect. The animal effects were assumed to be distributed as *N*(0, ***A*** *σ*_*a*_^2^), herd effects were assumed to be distributed as *N*(0, ***I*** *σ*_*herd*_^2^) and the residuals were assumed to be distributed as *N*(0, ***I*** *σ*_*e*_^2^), where **A** is the additive genetic relationships matrix, **I** is the identity matrix, *σ*_*a*_^2^ is the additive genetic variance, *σ*_*herd*_^2^ is the herd variance and, *σ*_*e*_^2^ is the residual variance. The statistical package ASReml [24] was used to perform the analyses. In the association analysis, the variance components were fixed to estimates obtained from model (1) without the SNP effect.

The heritability and the proportion of variance due to herd were calculated based on estimates from model (1) without the SNP effect. The heritability was calculated as

$$ {\mathrm{h}}^2=\frac{\upsigma_{\mathrm{a}}^2}{\left({\upsigma}_{\mathrm{a}}^2+{\upsigma}_{\mathrm{e}}^2\right)} $$

The proportion of variance due to differences among herds (h_herd_) was calculated as:

$$ {\mathrm{h}}_{\mathrm{h}\mathrm{erd}}=\frac{\upsigma_{\mathrm{h}\mathrm{erd}}^2}{\left({\upsigma}_{\mathrm{h}\mathrm{erd}}^2+{\upsigma}_{\mathrm{a}}^2+{\upsigma}_{\mathrm{e}}^2\right)} $$

To identify the SNPs that capture the genetic variation in β-LG content associated with the tail part of BTA11, a stepwise approach was adopted. For this purpose we zoomed in on the region from 100 to 110 Mb which contained 2,897 SNP of which 313 were non-polymorphic. After the first analysis, phenotypes were adjusted for the effect of the most significant SNP, which will be referred to as the lead SNP:

$$ {\mathrm{y}}_{\mathrm{klmno}}^{*}={\mathrm{y}}_{\mathrm{klmno}}-{\widehat{\mathrm{SNP}}}_{\mathrm{m}} $$

where y^*^_klmno_ is the β-LG content adjusted for the effect of the lead SNP genotype m. Estimated SNP genotype effects were obtained from model (1). Subsequently variance components were re-estimated for the adjusted phenotype (y^*^_klmno_) and the association study was repeated with variance components fixed at their new values. This procedure was repeated until *P* > 0.01 for the most significant SNP. The significant level of *P*-values = 0.01 equivalent to –log_10_(*P*-values) = 2 was chosen. False positive test were performed to check for multiple testing issue. In analogy to “Tag SNP”, i.e. a limited set of SNP that capture the genetic variation associated with a genomic region [25], we defined “Q-Tag SNP” as the set of SNP identified by the described procedure that capture the genetic variation of a chromosomal region. The β-LG content adjusted for the effect of Q-Tag SNP_1_ (lead SNP for the un-adjusted β-LG content) will be referred to as β-LG^1^, β-LG^2^ refers to β-LG^1^ adjusted for the effect of Q-Tag SNP_2_ (lead SNP for β-LG^1^) and so on.

Haplotypes were constructed based on Q-Tag SNP and effects of these haplotypes were estimated. The number of Q-Tag SNP that determine a haplotype was gradually increased by adding Q-Tag SNP in order of their number (i.e. Q-Tag SNP_1_, Q-Tag SNP_2_, Q-Tag SNP_3_ and so on). The association of haplotypes with β-LG content was estimated using the following animal model:

2 $$ \begin{array}{c}\hfill {\mathrm{y}}_{\mathrm{k}\mathrm{lmnop}}=\upmu +{\upbeta}_1\mathrm{di}{\mathrm{m}}_{\mathrm{k}\mathrm{lmnop}}+{\upbeta}_2{\mathrm{e}}^{-0.05\mathrm{di}{\mathrm{m}}_{\mathrm{k}\mathrm{lmnop}}}+{\upbeta}_3\mathrm{c}{\mathrm{a}}_{\mathrm{k}\mathrm{lmnop}}+{\upbeta}_4\mathrm{c}{\mathrm{a}}_{\mathrm{k}\mathrm{lmnop}}^2+\mathrm{seaso}{\mathrm{n}}_{\mathrm{k}}\hfill \\ {}\hfill +\mathrm{scod}{\mathrm{e}}_{\mathrm{l}}+\mathrm{haplo}{1}_{\mathrm{o}}+\mathrm{haplo}{2}_{\mathrm{p}}+\mathrm{anima}{\mathrm{l}}_{\mathrm{m}}+\mathrm{her}{\mathrm{d}}_{\mathrm{n}}+{\mathrm{e}}_{\mathrm{k}\mathrm{lmnop}}\hfill \end{array} $$

where the variables are as described for model (1) with the SNP effect being replaced by haplotype effects haplo1_o_ and haplo2_p_. haplo1_o_ is the effect of the first copy of an animal’s haplotype and haplo2_p_ is the effect of the second copy of an animal’s haplotype. The two haplotypes of an individual were randomly assigned to haplo1 or haplo2 and the design matrices of both haplotype effects were combined to estimate the effect of a particular haplotype. Haplotypes were modelled as random effects and assumed to be distributed as *N*(0, *Iσ*_*haplo*_^2^) where I is the identity matrix and σ^2^_haplo_ is the variation due to haplotypes.

Predicted values for the haplotype effects were calculated in ASReml [24] and a *t*-test was used to test if haplotype effects differed significantly. The haplotype effects were considered to be significantly different when *p* < 0.01. If haplotype effects did not differ, they were grouped and such a group of haplotypes will be referred to as “haplo-group”. The proportion of the phenotypic variance explained by haplotype groups was calculated based on model (2) as:

$$ {h}_{\mathrm{haplo}-\mathrm{group}}^2=\frac{\upsigma_{\mathrm{haplo}-\mathrm{group}}^2}{\left({\upsigma}_{\mathrm{haplo}-\mathrm{group}}^2+{\upsigma}_{\mathrm{a}}^2+{\upsigma}_{\mathrm{e}}^2\right)} $$

In order to determine the LD among the SNP between 75 Mb and 110 Mb of BTA11, the r^2^ was estimated using PLINK 1.07 [26]. By default the software is unphasing the data but an optional command was used to keep the phasing information for calculation of LD [27].

## Abbreviations

LD: Linkage disequilibrium
MAF: Minor allele frequency
*PAEP*: β-LG gene
QTL: Quantitative trait loci
SNP: Single nucleotide polymorphism
β-LG: beta-lactoglobulin
